# Policy Series: Impacts and Opportunities: Research and Policy Priorities at NIH

**DOI:** 10.1093/geroni/igaf122.756

**Published:** 2025-12-31

**Authors:** Patricia D’Antonio, Brian Lindberg

## Abstract

This symposium will explore the significant policy changes implemented by the current administration at the National Institutes of Health (NIH) and the National Institute on Aging (NIA), with a focus on their impact on the research community. We will examine how these shifts affect grant funding, application processes, and the overall research landscape.

